# The effects of ART on the dynamics of lipid profiles in Chinese Han HIV-infected patients: comparison between NRTI/NNRTI and NRTI/INSTI

**DOI:** 10.3389/fpubh.2023.1161503

**Published:** 2023-04-27

**Authors:** Shengnan Liu, Baozhu Wei, Wei Liang, Tielong Chen, Liping Deng, Min Zhao, Jing Wan

**Affiliations:** ^1^Department of Cardiology, Zhongnan Hospital of Wuhan University, Wuhan, China; ^2^School of Health Sciences, Wuhan University School of Health Sciences, Wuhan, China; ^3^Department of Infectious Diseases, Zhongnan Hospital of Wuhan University, Wuhan, China; ^4^Demonstration Center for Experimental Basic Medicine Education, School of Basic Medical Sciences, Wuhan University, Wuhan, China

**Keywords:** HIV, antiretroviral therapy, lipid profile, NNRTIs, INSTIs, generalized linear mixed-effects model

## Abstract

**Introduction:**

This article aimed to compare the prevalence of dyslipidemia and determine risk factors associated with lipid levels in a cohort of HIV-infected patients receiving two different antiretroviral therapy (ART) regimens, nucleoside reverse transcriptase inhibitor/non-nucleoside reverse transcriptase inhibitor (NRTI/NNRTI) and nucleoside reverse transcriptase inhibitor/integrase strand transfer inhibitor (NRTI/INSTI).

**Methods:**

This longitudinal study analyzed 633 HIV-infected patients with complete blood lipid profile records for at least 1 year at the ART clinic of Zhongnan Hospital of Wuhan University, China, from June 2018 to March 2021. Demographic and clinical data, including age, gender, body weight, height, current/former/non-smoker, current drinker, diabetes mellitus, hypertension, were extracted from electronic medical records. Laboratory tests included hematology, total cholesterol (TC), triglyceride (TG), low-density lipoprotein cholesterol (LDL-C), high-density lipoprotein cholesterol (HDL-C), Lipoprotein(a) and CD4 cell count. The observation duration of this study was a maximum of 33 months. Data comparisons were performed using the Chi-square test, Student’s *t*-test and Mann–Whitney *U* test. Generalized linear mixed-effects model (GLMM) and *value of p* < 0.05 were used to determine factors associated with serum lipid profiles.

**Results:**

In this study, the effect of the NNRTIs group on the lipid profile over time was mainly an increase in TC and HDL-C, while a decrease in TC/HDL-C and LDL/HDL-C. However, the INSTIs group had higher mean TC and lower HDL-C compared to the NNRTIs group, with significantly increased levels of TC, TG, HDL-C, and LDL-C. In the analysis of dyslipidemia rates, there were significant differences in the prevalence of abnormal TG and TC/HDL-C in HIV-infected patients receiving two different ART regimen groups during different follow-up periods. Dyslipidemia, defined as hypercholesterolemia, hypertriglyceridemia, and low HDL-C, was more prevalent in the INSTIs group, with a higher risk of developing hypertriglyceridemia and a higher TC/HDL-C ratio compared to the NNRTIs group. GLMM analysis suggested significantly higher TG values in the INSTIs group (estimated 0.36[0.10, 0.63], SE 0.14, *p* = 0.008) compared to the NNRTIs group, even after adjusting for other covariates. In addition, GLMM analysis also showed that age, gender, BMI, CD4 count, and ART duration were associated with dyslipidemia.

**Conclusion:**

In conclusion, treatment with both commonly-used ART regimens can increase the mean values of lipid profiles and the risk of dyslipidemia. The findings indicated that TG values were significantly higher in the INSTIs group than in HIV-infected patients receiving the NNRTIs regimens. Longitudinal TG values are independently associated with the clinical types of ART regimens.

**Clinical Trial Number**: ChiCTR2200059861.

## Introduction

Due to strong epidemiologic evidence, the benefits of increased application of combined antiretroviral therapy (ART) on life expectancy were well established (1). ART regimens substantially declined the morbidity and mortality in human immunodeficiency virus-infected patients (2, 3). However, people living with HIV/AIDS (PLWHA) frequently develop metabolic complications (lipid abnormalities and insulin resistance) and cardiovascular complications, despite the fact that current first-line ART regimens are associated with fewer severe adverse outcomes and fewer intolerable adverse effects than earlier antiretroviral therapies (4–7).

According to the Guidelines for the Use of Antiretroviral Agents in Adults and Adolescents with HIV, it was recommended that initial ART regimens for adults and adolescents generally consists of two nucleoside reverse transcriptase inhibitors (NRTIs) in combination with a third active antiretroviral drug from the following three drug classes: integrase strand transfer inhibitors (INSTIs), non-nucleoside reverse transcriptase inhibitors (NNRTIs), or protease inhibitors (PIs) (8). Recent findings indicated that treatment with ART regimens was associated with elevated levels of serum total cholesterol (TC), triglycerides (TG), low-density lipoprotein cholesterol (LDL-C), Lipoprotein(a), and low levels of high-density lipoprotein cholesterol (HDL-C) (8, 9).

Dyslipidemia, a major long-term adverse effects of current ART regimens, is not only a leading cause of cardiovascular disease (CVD), but also a key risk factor for preventable CVD-related mortality (4, 6). Despite the longer life expectancy, there was ample evidence that HIV-infected patients have an increased risk of CVD and significantly increased mortality from cardiovascular events compared with the general population. Therefore, selecting a more lipid-friendly regimen is imperative to improve dyslipidemia and to reduce CVD burdens in PLWHA (6, 10).

In HIV-infected patients receiving combination ART regimens, the effect of ART regimens on the lipid profile has been demonstrated, but uncertainty remains about the comparative effects of different ART regimens (8). Specifically, the effect on lipid profiles may vary among different ART regimens or different exposure durations to the same ART regimen. To provide an updated perspective on the comparative efficacy of different NRTIs-based ART regimens on lipid profiles, an observational cohort study was conducted to estimate the effects of NNRTIs or INSTIs regimens and the exposure durations to those regimens on lipid profile alterations, and to explore factors associated with serum lipid profiles in HIV-infected patients.

## Methods

### Study design

A monocentric longitudinal observational cohort study.

### Study setting

This longitudinal cohort study was conducted in an outpatient cohort of ART clinic of Zhongnan Hospital of Wuhan University, from June 2018 to March 2021, which provides HIV/AIDS interventions including free diagnosis, treatment and monitoring.

### Source population

All HIV-infected patients receiving HAART at the ART Clinic of Zhongnan Hospital of Wuhan University from June 2018 to March 2021.

### Study population

All HIV-infected patients receiving NRTIs in combination with NNRTIs, NRTIs in combination with INSTIs for at least 1 year at the ART Clinic.

### Inclusion criteria and exclusion criteria

Inclusion criteria were as follows: (1) HIV-infected patients participating HIV/AIDS prevention treatment control programs at the ART Clinic; (2) follow-up were conducted at six-month intervals on average; (3) having at least two follow-up measurements of blood lipids; (4) receiving first-line ART regimens consisted of 2 NRTIs in combination with 1 NNRTI or 2 NRTIs in combination with 1 INSTI; (5) HIV-infected patients who constantly and regularly maintained the assigned treatment regimen at least during the study period.

Exclusion criteria were as follows: (1) Those who has severe liver, kidney, heart, brain and other dysfunctions; (2) Presence of viral hepatitis, active syphilis, tuberculosis, autoimmune diseases and other active infectious diseases; (3) Women who are known to be pregnant, lactating, or intending to become pregnant during the study; (4) Those who modified ART medication between available lipid measurements; (5) Having no follow-up testing for blood lipids within 6 months of ART initiation; (6) Receiving protease inhibitors or other ART regimens; and (7) Those who have poor compliance and cannot cooperate with treatment and intervention (Appendix Figure 1).

### Determination of sample size

A census sampling was used, in which 633 eligible patients visiting the ART clinic were included for this study (Appendix Figure 1).

### Study parameters

*Exposure*: ART regimens consisted of 2 NRTIs in combination with 1 NNRTI or 2 NRTIs in combination with 1 INSTI.

*Independent Variables*: Potential confounders of lipid profile such as age, gender, weight, body mass index, drinking habit, smoking habit, hypertension, fasting plasma glucose, duration on HAART, CD4 count, T lymphocyte count and HIV-1 viral load as independent variables were included in the final model.

*Dependent Variable*: Total cholesterol (TC), triglyceride (TG), low-density lipoprotein cholesterol (LDL-C), high-density lipoprotein cholesterol (HDL-C), Lipoprotein(a), TC/HDL-C, TG/HDL-C, and LDL-C/HDL-C level.

*Outcomes*: Baseline means were estimated first and then the follow-up means subtracted for the baseline were estimated to account for differences in mean values of lipid profiles among HIV patients by type of ART regimen at different follow-up periods and baseline examinations. Prevalence of lipid abnormalities between different ART regimens during each follow-up period. Generalized linear mixed-effects model (GLMM) was used to identify factors affecting serum lipid profiles.

### Data collection

Data were extracted from an electronic medical records system, including demographic, clinical characteristics, and laboratory results. Demographic and clinical data comprehensively included age, gender, body weight, height, lifestyle behaviors (smoking and drinking), medical history (including hypertension, diabetes), duration of ART use and types of ART regimens. Laboratory tests included hematology, total cholesterol (TC), triglyceride (TG), low-density lipoprotein cholesterol (LDL-C), high-density lipoprotein cholesterol (HDL-C), Lipoprotein(a), and CD4 cell count. All patients were assigned unique identification numbers to anonymize data. After initiation of ART, patients were evaluated at six-month intervals with the above-described laboratory tests repeated. The observation period for this study was a maximum of 33 months. Data were carefully reviewed by two independent researchers. Discrepancies between the reviewers were resolved by discussion or a third researcher. All methods were performed in accordance with relevant guidelines and regulations.

### Anthropometric measurements and blood sample collection

Weight was categorized according to BMI as underweight (less than 18.5 kg/m^2^), normal (18.5–24.9 kg/m^2^), overweight (25–29.9 kg/m^2^), and obese (equal to or greater than 30 kg/m^2^) (11). According to the National Cholesterol Education Program, Adult Treatment Panel III (NCEP-ATP III) guidelines, hypercholesterolemia is defined as total cholesterol ≥ 200 mg/dl (≥ 5.18 mmol/l), hypertriglyceridemia is defined as triglycerides ≥ 150 mg/dl (≥ 1.70 mmol/l), low HDL cholesterol as HDL-C ≤ 40 mg/dl (≤1.04 mmol/l), and high LDL cholesterol as LDL-C ≥ 130 mg/dl (≥ 3.37 mmol/l). Hypercholesterolemia, hypertriglyceridemia, and low HDL cholesterol were considered dyslipidemia (12). In addition, elevated Lp(a) levels were defined as Lp(a) ≥ 30 mg/dl (13, 14).

### Statistical analysis

All statistical analyses were performed using RStudio (version 2021.09.1.0) and Graphpad Prism (version 8.1 for Windows). All tests were two-tailed and *p* values less than 5% were considered statistically significant. Measurement data were tested using Levene’s method for homogeneity of variance and Shapiro–Wilk (S–W) test for normal distribution. Categorical variables were presented as frequency rates and percentages (%), and Chi-squared tests were used to compare the proportions of categorical variables. Fisher’s exact test was used when limited data were observed. When the measurement data were normally distributed, the mean ± standard deviation (mean ± SD) was used, and the independent group t-test was used to compare the means of continuous variables. Non-normally distributed measurement data were expressed as medians (interquartile range [IQR]), and Mann–Whitney *U* test was used to test for significant differences between groups, as appropriate. Due to repeat measurements of blood lipids profiles, generalized linear mixed-effects models (GLMM) were used to determine factors associated with each serum lipid profile across all study participants, using coefficient estimates with 95% confidence intervals (CIs). To accommodate unbalanced, unequally spaced observations, the Generalized additive mixed models (GAMM) were used to present the effects of different ART regimens on triglyceride over time after adjusting for covariates of age, gender and BMI. In addition, a sensitivity analysis of associated factors for lipidemia by generalized estimating equation (GEE).

### Ethical consideration

The study was approved by the Institutional Review Board of College of Zhongnan Hospital of Wuhan University (No. 2022122 K), and written informed consent was obtained from the study participants. All research procedures were performed in accordance with the approved guidelines and regulations according to the criteria of the Declaration of Helsinki. The Chinese Clinical Trial Registry number of this work is ChiCTR2200059861.^1^

## Results

### Patient characteristics

This longitudinal cohort study analyzed 633 HIV-infected patients receiving routine HAART treatment and monitoring at the ART Clinic. Of these, 422 patients (64%) were treated with the NNRTIs regimens, and 211 patients (22%) were treated with the INSTIs regimens. The total follow-up time was 10,794 patient-months (900 patient-years), and the median follow-up duration was 17 months. The mean (± standard deviation [SD]) age of all patients was 35.8 ± 14.8 years, ranging from 11 to 83 years, and 31 patients (4.9%) were female. The average BMI of HIV-infected patients treated with different ART regimens were similar (*p* = 0.919). In addition, there was no difference at baseline in the percentage of smokers, drinkers, and patients with hypertension between the two ART treatment groups (*p* > 0.05 for all). Among all HIV-infected patients participating in this study, 14 HIV patients had the previous history of gout complications, 5 patients had the history of cerebrovascular disease, and only 2 patients had the history of coronary heart disease, and other patients denied baseline comorbidities. It should be noted that complete baseline lipid data were analyzed for only 546 patients, and lipid profiles were not obtained from the remaining 87 patients within the week prior to ART use. There were no significant differences in the baseline characteristics of HIV-infected patients at each ART treatment group (*p* > 0.05 for all; Table 1; Appendix Table 1).

**Table 1:** Baseline characteristics of HIV patients before receiving ART by types of ART regimen.

Variables	Normal range	All patients (*n* = 633)	NNRTIs (*n* = 422)	INSTIs (*n* = 211)	*p* Value
Age, years, median [IQR]	NA	31 [25–44]	31 [24–44]	33 [26–45]	0.059
Gender, Female, *n* (%)	NA	31 (4.9)	24 (5.7)	7 (3.3)	0.193
Weight, (kg), median [IQR]	NA	64 [59–71]	64 [60–70]	65 [57–72]	0.725
**BMI**, (kg/m^2^), median [IQR]	18.5–24.9	21.5 [19.7–23.4]	21.5 [19.6–23.6]	21.6 [20.0–23.3]	0.919
Underweight, *n* (%)	<18.5	45 (7.1)	25 (5.9)	20 (9.5)	
Normal, *n* (%)	18.5–24.9	273 (43.1)	164 (38.9)	109 (51.7)	
Overweight, *n* (%)	25–29.9	53 (8.4)	32 (7.6)	21 (10.0)	
Obese, *n* (%)	>30	8 (1.3)	5 (1.2)	3 (1.4)	
Lack of BMI data/unknown, *n* (%)	NA	254 (40.1)	196 (46.4)	58 (27.5)	
Drinking habit, *n* (%)	NA	105 (16.6)	71 (16.8)	34 (16.1)	0.821
Smoking habit, *n* (%)	NA	116 (18.3)	73 (17.3)	43 (20.4)	0.383
Hypertension, *n* (%)	NA	31 (4.9)	18 (4.3)	13 (6.2)	0.297
Laboratory findings		All patients (n = 546)	NNRTIs (n = 377)	INSTIs (n = 169)	
**FPG**, mmol/L, median [IQR]	3.9–6.1	5.13 [4.78–5.55]	5.09 [4.79–5.54]	5.21 [4.76–5.57]	0.649
IFG, *n* (%)	5.6–6.9	103 (18.9)	67 (17.8)	36 (21.3)	0.539
Diabetes, *n* (%)	≥7.0	17 (3.1)	14 (3.7)	3 (1.8)	0.228
**TC**, mmol/L, median [IQR]	<5.18	3.88 [3.43–4.47]	3.86 [3.44–4.45]	3.90 [3.42–4.55]	0.744
Normal, *n* (%)	<200 mg/dl; <5.18 mmol/l	502 (91.9)	348 (92.3)	154 (91.1)	
Borderline-high, *n* (%)	200-239 mg/dl; 5.18–6.21 mmol/l	37 (6.8)	23 (6.1)	14 (8.3)	0.467
High, *n* (%)	≥240 mg/dl; ≥6.21 mmol/l	7 (1.3)	6 (1.6)	1 (0.6)	
**TG**, mmol/L, median [IQR]	<1.70	1.14 [0.85–1.61]	1.13 [0.84–1.57]	1.20 [0.87–1.72]	0.152
Normal, *n* (%)	<150 mg/dl; <1.70 mmol/l	427 (78.2)	301 (79.8)	126 (74.6)	
Borderline-high, *n* (%)	150-199 mg/dl;1.70–2.26 mmol/l	66 (12.1)	44 (11.7)	22 (13.0)	0.293
High, *n* (%)	≥200 mg/dl; ≥2.26 mmol/l	53 (9.7)	32 (8.5)	21 (12.4)	
**HDL-C**, mmol/L, median [IQR]	>1.04	0.98 [0.84–1.12]	0.98 [0.85–1.12]	0.97 [0.80–1.12]	0.443
Low, *n* (%)	<40 mg/dl; <1.04 mmol/l	338 (61.9)	232 (61.5)	106 (62.7)	
Borderline-high, *n* (%)	40-59 mg/dl; 1.04–1.55 mmol/l	195 (35.7)	136 (36.1)	59 (34.9)	0.967
High, *n* (%)	≥60 mg/dl; ≥1.55 mmol/l	13 (2.4)	9 (2.4)	4(2.4)	
**LDL-C**, mmol/L, median [IQR]	<3.37	2.44 [1.97–2.85]	2.45 [2.01–2.83]	2.42 [1.89–2.88]	0.439
Normal, *n* (%)	<130 mg/dl; <3.37 mmol/l	490 (89.7)	340 (90.2)	150 (88.8)	
Borderline-high, *n* (%)	130-159 mg/dl; 3.37–4.14 mmol/l	43 (7.9)	27 (7.2)	16 (9.5)	0.588
High, *n* (%)	≥160 mg/dl; ≥4.14 mmol/l	13 (2.4)	10 (2.7)	3 (1.8)	
Lp(a), mg/dL, median [IQR]	<30	7.59 [4.63–15.01]	7.48 [4.64–15.75]	7.98 [4.44–13.09]	0.581
Normal, *n* (%)	<30 mg/dl	492 (90.1)	337 (89.4)	155 (91.7)	
Abnormal, *n* (%)	≥30 mg/dl	54 (9.9)	40 (10.6)	14 (8.3)	0.400
CD4 count, cells/mm^3^, median [IQR]	550–1,440	274 ± 153	284 ± 147	257 ± 163	0.410
Absolute T lymphocyte count, cells/mm^3^, median [IQR]	955–2,860	1,232 [817–1701]	1,228 [879–1764]	1,247 [718–1,454]	0.246

NA = not available. *p* values < 0.05 are written in italics.

Values shown are mean ± SD, median (interquartile range [IQR]) or *n* (%). *p* values were calculated by Chi-squared test, Fisher’s exact test, *t* test, or Mann–Whitney *U* test, as appropriate.

HIV, human immune deficiency virus; ART, antiretroviral therapy, n number; IQR, Inter Quartile Range; SD, standard deviation, % percentage; NNRTIs, non-nucleoside reverse transcriptase inhibitor; INSTIs, integrase strand transfer inhibitors; BMI, body mass index; FPG, fasting plasma glucose; IFG, impaired fasting glucose; TC, total cholesterol; TG, triglyceride; HDL-C, high-density lipoprotein-cholesterol; LDL-C, low-density lipoprotein-cholesterol; Lipoprotein(a), Lp(a).

### Mean lipid levels

**Figure 1:**
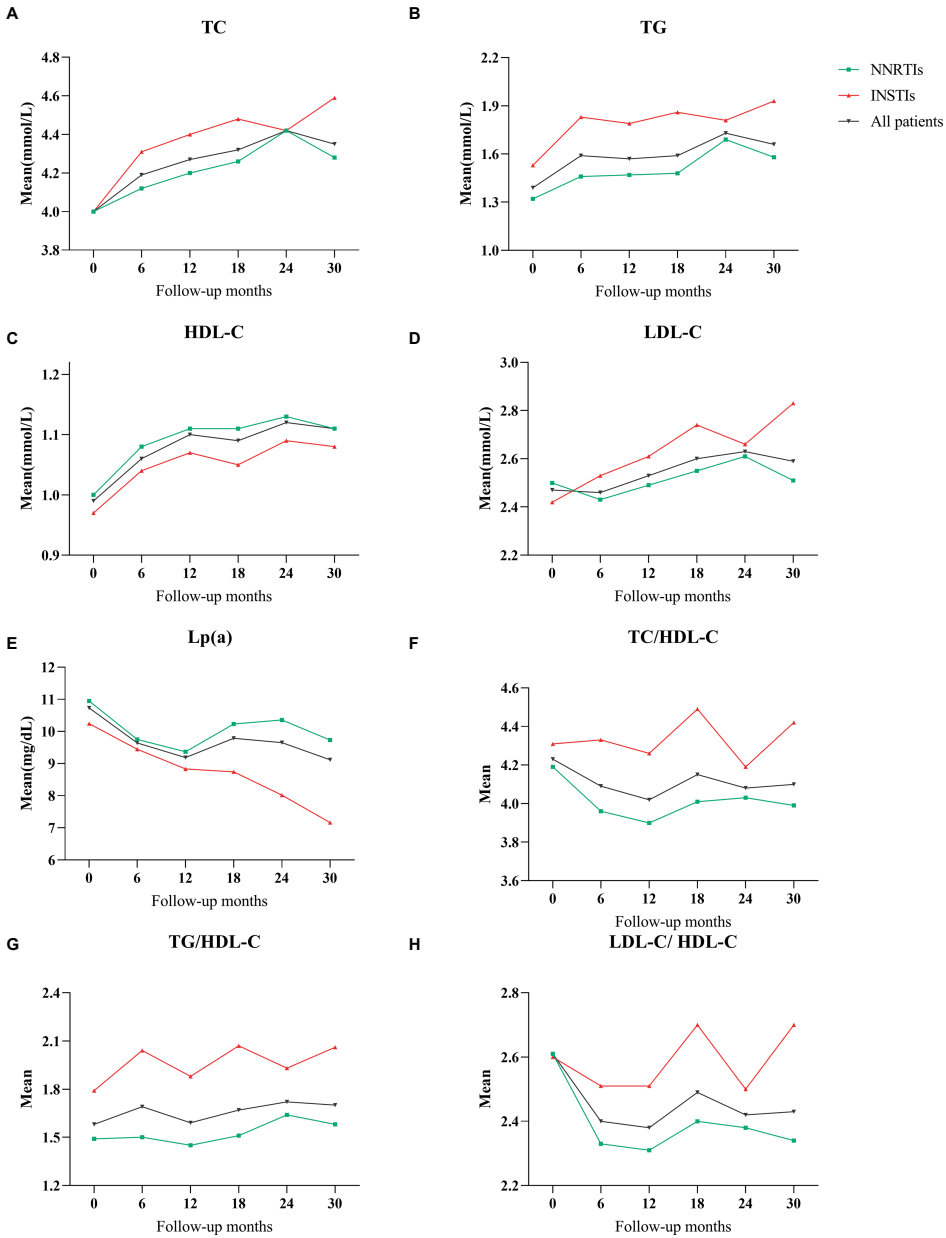
**(A)** The mean values of total cholesterol during follow-up period with NNRTIs vs. INSTIs. **(B)** Triglycerides throughout the follow-up period. **(C)** HDL-cholesterol. **(D)** LDL-cholesterol. **(E)** Lipoprotein(a). **(F)** TC/HDL-C ratio. **(G)** TG/HDL-C ratio. **(H)** LDL-C/HDL-C ratio.

According to the type of ART regimens, mean differences of lipid profile at different follow-up periods with baseline data of HIV-infected patients were compared. The results were summarized in Table 2, and the change trends over time were presented in Figure 1. In patients treated with NNRTIs, the mean values of TC and HDL-C over time were significantly higher during the follow-up periods than those at the baseline, whereas the mean values of TC/HDL-C as well as LDL-C/HDL-C were significantly lower during the follow-up periods (*p* < 0.05 for all; Figure 1). Atherogenic dyslipidemia, including low HDL-C and high TC, were main cardiovascular risk factors in HIV-infected patients (6, 15, 16). Compared with the mean values of the baseline lipid profile, the mean values of TC, HDL-C and LDL-C were significantly increased in the INSTIs-treated group (*p* < 0.05 for all).

**Table 2:** Comparison of blood lipid levels between baseline data with different follow-up periods by types of ART regimen.

	Baseline	At 6 months	*p*-value	At 12 months	*P*-value	At 18 months	*p*-value	At 24 months	*p*-value	At 30 months	*p*-value
*A. NNRTIs Group, mean ± SD*											
TC, mmol/L	4.00 ± 0.83	4.12 ± 0.84	0.063	4.20 ± 0.91	*0.004*	4.26 ± 1.01	*0.001*	4.42 ± 1.02	*<0.001*	4.28 ± 1.07	*0.006*
TG, mmol/L	1.32 ± 0.76	1.46 ± 1.02	*0.039*	1.47 ± 0.91	*0.029*	1.48 ± 1.35	0.074	1.69 ± 1.47	*<0.001*	1.58 ± 1.66	*0.026*
HDL-C, mmol/L	1.00 ± 0.25	1.08 ± 0.24	*<0.001*	1.11 ± 0.25	*<0.001*	1.11 ± 0.27	*<0.001*	1.13 ± 0.25	*<0.001*	1.11 ± 0.25	*<0.001*
LDL-C, mmol/L	2.50 ± 0.74	2.43 ± 0.66	0.216	2.49 ± 0.72	0.859	2.55 ± 0.81	0.463	2.61 ± 0.73	0.122	2.51 ± 0.70	0.850
Lp(a), mg/dL	10.95 ± 9.40	9.75 ± 8.93	0.098	9.37 ± 8.48	*0.032*	10.23 ± 9.87	0.396	10.36 ± 8.44	0.579	9.73 ± 9.56	*0.252*
TC/HDL-C	4.19 ± 1.14	3.96 ± 1.03	*0.005*	3.90 ± 0.93	*0.001*	4.01 ± 1.46	*0.013*	4.03 ± 1.03	0.150	3.99 ± 1.29	*0.*127
TG/HDL-C	1.49 ± 1.16	1.50 ± 1.32	0.883	1.45 ± 1.14	0.694	1.51 ± 1.74	0.856	1.64 ± 1.56	0.251	1.58 ± 2.06	0.541
LDL-C/ HDL-C	2.61 ± 0.91	2.33 ± 0.72	*<0.001*	2.31 ± 0.70	*<0.001*	2.40 ± 0.99	*0.009*	2.38 ± 0.67	*0.006*	2.34 ± 0.72	*0.006*
*B. INSTIs Group, mean ± SD*											
	Baseline	At 6 months	*P*-value	At 12 months	*P*-value	At 18 months	*P*-value	At 24 months	*P*-value	At 30 months	*P*-value
TC, mmol/L	4.00 ± 0.88	4.31 ± 0.87	*0.001*	4.40 ± 0.87	*<0.001*	4.48 ± 1.01	*<0.001*	4.42 ± 0.96	*0.003*	4.59 ± 1.05	*0.001*
TG, mmol/L	1.53 ± 1.24	1.83 ± 1.72	0.067	1.79 ± 1.45	0.087	1.86 ± 1.73	*0.015*	1.81 ± 1.54	0.165	1.93 ± 1.26	*0.038*
HDL-C, mmol/L	0.97 ± 0.25	1.04 ± 0.22	*0.015*	1.07 ± 0.24	*0.001*	1.05 ± 0.24	*0.017*	1.09 ± 0.25	*0.002*	1.08 ± 0.21	*0.025*
LDL-C, mmol/L	2.42 ± 0.79	2.53 ± 0.70	0.187	2.61 ± 0.68	*0.029*	2.74 ± 0.72	*0.002*	2.66 ± 0.76	*0.043*	2.83 ± 0.98	*0.009*
Lp(a), mg/dL	10.24 ± 8.36	9.45 ± 7.92	0.381	8.83 ± 7.76	0.143	8.74 ± 8.10	0.178	8.02 ± 7.79	0.106	7.16 ± 6.74	*0.*055
TC/HDL-C	4.31 ± 1.25	4.33 ± 1.26	0.921	4.26 ± 1.11	0.703	4.49 ± 1.74	0.345	4.19 ± 1.16	0.526	4.42 ± 1.30	0.643
TG/HDL-C	1.79 ± 1.78	2.04 ± 2.76	0.318	1.88 ± 1.90	0.646	2.07 ± 2.83	0.332	1.93 ± 2.22	0.623	2.06 ± 1.82	0.413
LDL-C/ HDL-C	2.60 ± 1.00	2.51 ± 0.80	0.336	2.51 ± 0.73	0.357	2.70 ± 0.84	0.417	2.50 ± 0.75	0.501	2.70 ± 1.00	0.605
*C. All patients, mean ± SD*											
	Baseline	At 6 months	*P*-value	At 12 months	*P*-value	At 18 months	*P*-value	At 24 months	*P*-value	At 30 months	*P*-value
TC, mmol/L	4.00 ± 0.85	4.19 ± 0.85	*<0.001*	4.27 ± 0.90	*<0.001*	4.32 ± 1.01	*<0.001*	4.42 ± 1.00	*<0.001*	4.35 ± 1.07	*0.001*
TG, mmol/L	1.39 ± 0.94	1.59 ± 1.13	*0.004*	1.57 ± 1.11	*0.006*	1.59 ± 1.48	*0.015*	1.73 ± 1.48	*<0.001*	1.66 ± 1.58	0.*009*
HDL-C, mmol/L	0.99 ± 0.25	1.06 ± 0.24	*<0.001*	1.10 ± 0.25	*<0.001*	1.09 ± 0.26	*<0.001*	1.12 ± 0.25	*<0.001*	1.10 ± 0.24	*<0.001*
LDL-C, mmol/L	2.47 ± 0.76	2.46 ± 0.68	0.846	2.53 ± 0.71	0.269	2.60 ± 0.79	*0.020*	2.63 ± 0.74	*0.015*	2.59 ± 0.78	0.107
Lp(a), mg/dL	10.73 ± 9.08	9.64 ± 8.58	0.058	9.19 ± 8.25	*0.009*	9.79 ± 9.40	*0.*171	9.65 ± 9.75	*0.026*	9.12 ± 9.02	*0.*071
TC/HDL-C	4.23 ± 1.18	4.09 ± 1.13	*0.049*	4.02 ± 1.01	*0.003*	4.15 ± 1.56	0.423	4.08 ± 1.07	0.114	4.10 ± 1.30	0.249
TG/HDL-C	1.58 ± 1.39	1.69 ± 1.56	0.301	1.59 ± 1.44	0.921	1.67 ± 2.13	0.460	1.72 ± 1.83	0.275	1.70 ± 2.01	0.421
LDL-C/ HDL-C	2.61 ± 0.94	2.40 ± 0.75	*<0.001*	2.38 ± 0.72	*<0.001*	2.49 ± 0.96	0.079	2.42 ± 0.70	*0.009*	2.43 ± 0.81	0.*041*

*p* values are all against baseline; *p* values < 0.05 are written in italics.

Values shown are mean ± SD. *p* values were calculated by Student’s *t*-test, or Wilcoxon test, as appropriate.

ART, antiretroviral therapy; SD, standard deviation; NNRTIs, non-nucleoside reverse transcriptase inhibitor; INSTIs, integrase strand transfer inhibitors; TC total cholesterol; TG, triglyceride; HDL-C, high-density lipoprotein-cholesterol; LDL-C, low-density lipoprotein-cholesterol; Lipoprotein(a), Lp(a); TC/HDL-C ratio, total cholesterol/high-density lipoprotein-cholesterol ratio.

For all patients on the ART regimens, the mean values of TC, TG and HDL-C showed a significant increase during the follow-up periods compared with the baseline data (*p* < 0.05 for all). In addition, even though there were no statistically significant differences, the mean values of TG/HDL-C were slightly higher than baseline in all HIV-infected patients during the follow-up periods (Figure 1).

### Prevalence of dyslipidemia

**Figure 2:**
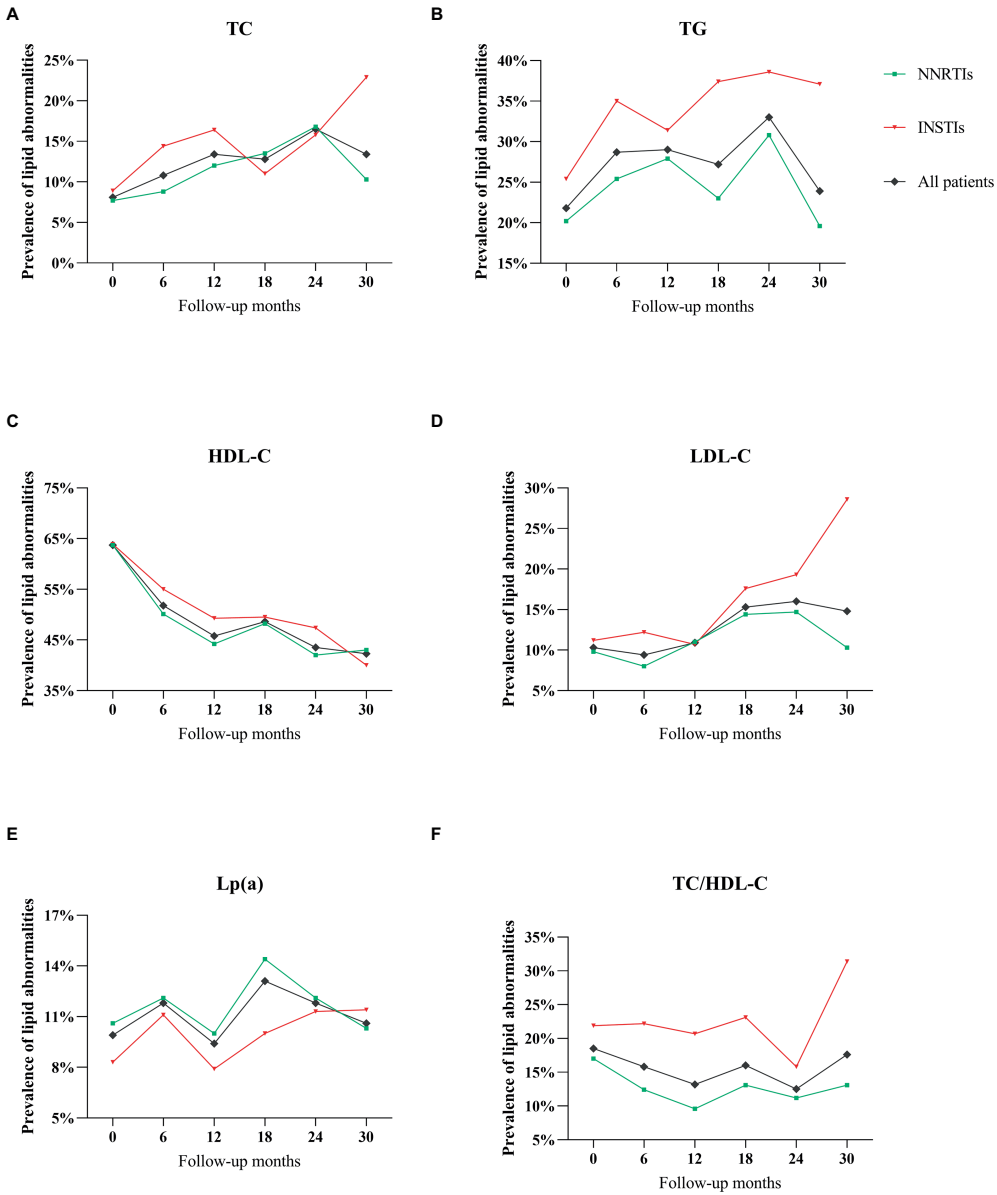
**(A)** The prevalence of abnormal total cholesterol during follow-up period with NNRTIs vs. INSTIs. **(B)** Triglycerides throughout the follow-up period. **(C)** HDL-cholesterol. **(D)** LDL-cholesterol. **(E)** Lipoprotein(a). **(F)** TC/HDL-C ratio.

The prevalence of lipid abnormalities at baseline and during follow-up periods for the two different ART regimen was reported in Table 3 and Figure 2. In the analysis of dyslipidemia rates for all patients, the proportion of abnormal TC, TG and LDL-C were slightly higher overtime from baseline to 30 months, whereas the prevalence of abnormal HDL-C as well as TC/HDL-C were lower during the follow-up periods (Figure 2).

**Table 3:** Prevalence of lipid abnormalities between baseline data with different follow-up periods.

	Baseline (*n* = 546)	At 6 months (*n* = 519)	At 12 months (*n* = 441)	At 18 months (*n* = 313)	At 24 months (*n* = 200)	At 30 months (*n* = 142)
*A. TC, ≥ 5.18 mmol/l; ≥ 200 mg/dl*						
NNRTIs Group	29 (7.7)	30 (8.8)	36 (12.0)	30 (13.5)	24 (16.8)	11 (10.3)
INSTIs Group	15 (8.9)	26 (14.4)	23 (16.4)	10 (11.0)	9 (15.8)	8 (22.9)
*P-*value	0.639	0.051	0.199	0.544	0.864	0.083
All patients	44 (8.1)	56 (10.8)	59 (13.4)	40 (12.8)	33 (16.5)	19 (13.4)
*B. TG, ≥ 1.70 mmol/l; ≥ 150 mg/dl*						
	Baseline	At 6 months	At 12 months	At 18 months	At 24 months	At 30 months
NNRTIs Group	76 (20.2)	86 (25.4)	84 (27.9)	51 (23.0)	44 (30.8)	21(19.6)
INSTIs Group	43 (25.4)	63 (35.0)	44 (31.4)	34 (37.4)	22 (38.6)	13(37.1)
*P*-value	0.167	***0.021***	0.448	***0.009***	0.288	***0.035***
All patients	119 (21.8)	149 (28.7)	128 (29.0)	85 (27.2)	66 (33.0)	34(23.9)
*C. HDL-C, ≤ 1.04 mmol/l; < 40 mg/dl*						
	Baseline	At 6 months	At 12 months	At 18 months	At 24 months	At 30 months
NNRTIs Group	240 (63.7)	170 (50.1)	133 (44.2)	107 (48.2)	60 (42.0)	46 (43.0)
INSTIs Group	108 (63.9)	99 (55.0)	69 (49.3)	45 (49.5)	27 (47.4)	14 (40.0)
*P-*value	0.956	0.292	0.317	0.840	0.486	0.756
All patients	348 (63.7)	269 (51.8)	202 (45.8)	152 (48.6)	87 (43.5)	60 (42.3)
*D. LDL-C, ≥ 3.37 mmol/l; ≥ 130 mg/dl*						
	Baseline	At 6 months	At 12 months	At 18 months	At 24 months	At 30 months
NNRTIs Group	37 (9.8)	27 (8.0)	33 (11.0)	32 (14.4)	21 (14.7)	11 (10.3)
INSTIs Group	19 (11.2)	22 (12.2)	15 (10.7)	16 (17.6)	11 (19.3)	10 (28.6)
*P-*value	0.611	0.114	0.938	0.480	0.422	***0.008***
All patients	56 (10.3)	49 (9.4)	48 (10.9)	48 (15.3)	32 (16.0)	21 (14.8)
*E. Lp(a), ≥30 mg/dl*						
	Baseline	At 6 months	At 12 months	At 18 months	At 24 months	At 30 months
NNRTIs Group	40 (10.6)	41 (12.1)	30 (10.0)	32 (14.4)	14 (12.1)	11 (10.3)
INSTIs Group	14 (8.3)	20 (11.1)	11 (7.9)	9 (10.0)	6 (11.3)	4 (11.4)
*P-*value	0.400	0.741	0.478	0.296	0.889	0.849
All patients	54 (9.9)	61 (11.8)	41 (9.4)	41 (13.1)	20 (11.8)	15 (10.6)
*F. TC/HDL-C, >5*						
	Baseline	At 6 months	At 12 months	At 18 months	At 24 months	At 30 months
NNRTIs Group	64 (17.0)	42 (12.4)	29 (9.6)	29 (13.1)	16 (11.2)	14 (13.1)
INSTIs Group	37 (21.9)	40 (22.2)	29 (20.7)	21 (23.1)	9 (15.8)	11 (31.4)
*P*-value	0.171	***0.003***	***0.001***	***0.028***	0.374	***0.013***
All patients	101 (18.5)	82 (15.8)	58 (13.2)	50 (16.0)	25 (12.5)	25 (17.6)

NA = not available. *p* values < 0.05 are written in italics.

Values shown are (%). *p* values were calculated by Chi-squared test, Fisher’s exact test, as appropriate.

ART, antiretroviral therapy; %, percentage, NNRTIs, non-nucleoside reverse transcriptase inhibitor; INSTIs, integrase strand transfer inhibitors; TC, total cholesterol; TG, triglyceride; HDL-C, high-density lipoprotein-cholesterol; LDL-C, low-density lipoprotein-cholesterol; Lp(a), Lipoprotein(a); TC/HDL-C ratio, total cholesterol/high-density lipoprotein-cholesterol ratio.

No significant difference in the prevalence of abnormal TC, HDL-C and Lp(a) during follow-up periods were observed between different ART regimens (*p* > 0.05 for all), while there was a significant difference in the prevalence of abnormal TG, LDL-C and TC/HDL-C in HIV-infected patients receiving different ART regimens during different follow-up periods (Table 3). Notably, HIV-infected patients on INSTIs regimen were more likely to exhibit the prevalence of TG compared with those on NNRTIs regimens (*p* < 0.05; Table 3; Figure 2).

### Factors associated with elevated lipids

Generalized linear mixed-effects models (GLMM) were applied to assess factors associated with each serum lipid profile (Table 4; Appendix Table 2). The Akaike information criteria and the Bayesian information criteria were used to evaluate and compare the different model fits (Appendix Table 3). Multivariable analyses for lipid profile were adjusted for age, gender, body mass index, drinking habit, smoking habit, hypertension, CD4 count, T lymphocyte count and HIV-1 viral load. Fasting plasma glucose was included as a time-varying covariates. In addition, the duration on HAART, ART regimens and an interaction term between duration on HAART and type of ART regimens were included to the mixed effect models. After full adjustment for other covariates, GLMM analysis showed no statistically significant interaction between duration on ART and type of ART regimens for each serum lipid profile (*p* for interaction > 0.05; Appendix Table 2).

**Table 4:** Estimates of the generalized linear mixed-effects model (GLMM) for lipidemia over time.

	TC			TG			HDL-C		
Fixed Effects	Estimate	Std. Error	*p* values	Estimate	Std. Error	*p* values	Estimate	Std. Error	*p* values
Time	0.02	0.01	***0.032***	0.02	0.01	***0.039***	0.00	0.00	0.397
INSTIs vs. NNRTIs	0.20	0.12	0.110	0.36	0.14	***0.008***	−0.02	0.04	0.658
Time: INSTIs vs. NNRTIs	0.01	0.01	0.553	−0.01	0.01	0.494	0.00	0.00	0.907
	LDL-C			Lp(a)			TC/HDL-C		
Fixed Effects	Estimate	Std. Error	*p* values	Estimate	Std. Error	*p* values	Estimate	Std. Error	*p* values
Time	0.01	0.01	0.111	−0.05	0.07	0.457	0.01	0.01	0.495
INSTIs vs. NNRTIs	0.06	0.10	0.563	−2.18	2.14	0.309	0.27	0.15	0.066
Time: INSTIs vs. NNRTIs	0.01	0.01	0.122	0.10	0.09	0.247	0.01	0.01	0.639

NA = not available. *p* values < 0.05 are written in italics.

NNRTIs, non-nucleoside reverse transcriptase inhibitor; PIs, protease inhibitors; INSTIs, integrase strand transfer inhibitors; BMI, body mass index; TC, total cholesterol; TG, triglyceride; HDL-C, high-density lipoprotein-cholesterol; LDL-C, low-density lipoprotein-cholesterol; Lp(a), Lipoprotein(a).

In GLMM analysis adjusted for various confounders, age was significantly associated with higher TC and higher TG. Compared to the NNRTIs group, a significantly higher risk of TG elevation was observed in the INSTIs group (estimate 0.36[0.10, 0.63], SE 0.14, *p* = 0.008). In addition, high BMI were associated with increased TG and decreased HDL-C. Conversely, lower CD4 count were associated with a raised HDL-C. It was also worth noting that HIV-infected patients with lower T lymphocyte count were more likely to have lower TC and TG. Furthermore, duration on ART was also an independent factor for elevated TC and TG (Table 4; Appendix Table 2).

The results of GLMM model revealed that FPG < 7.0 and smoking habit were associated with significantly higher LDL-C and Lp(a) levels. In addition, a significantly higher risk of Lp(a) elevation was observed in the older HIV-infected patients. Regarding TC/HDL-C, those HIV-infected patients with higher BMI were more likely to have elevated TC/HDL-C as compared with lower BMI. Moreover, older age, smoking habit, and increased CD4 count and T lymphocyte count were associated with increased TC/HDL-C (Appendix Table 2).

### Sensitivity analyses of Lipidemia

In order to avoid potential attrition bias, the lipid profile analyses restricted to HIV-infected patients with 24 months of follow-up was repeated by generalized estimating equation (GEE). The result of sensitivity analyses further supported similar lipid profile changes as the main analysis (Appendix Table 2). Compared with HIV-infected patients receiving the NNRTIs regimens, TG values were significantly higher in the INSTIs group (Exp(β) 1.67[1.05, 2.67], *p* = 0.031; Appendix Table 4).

### Association between ART regimens and triglyceride

**Figure 3:**
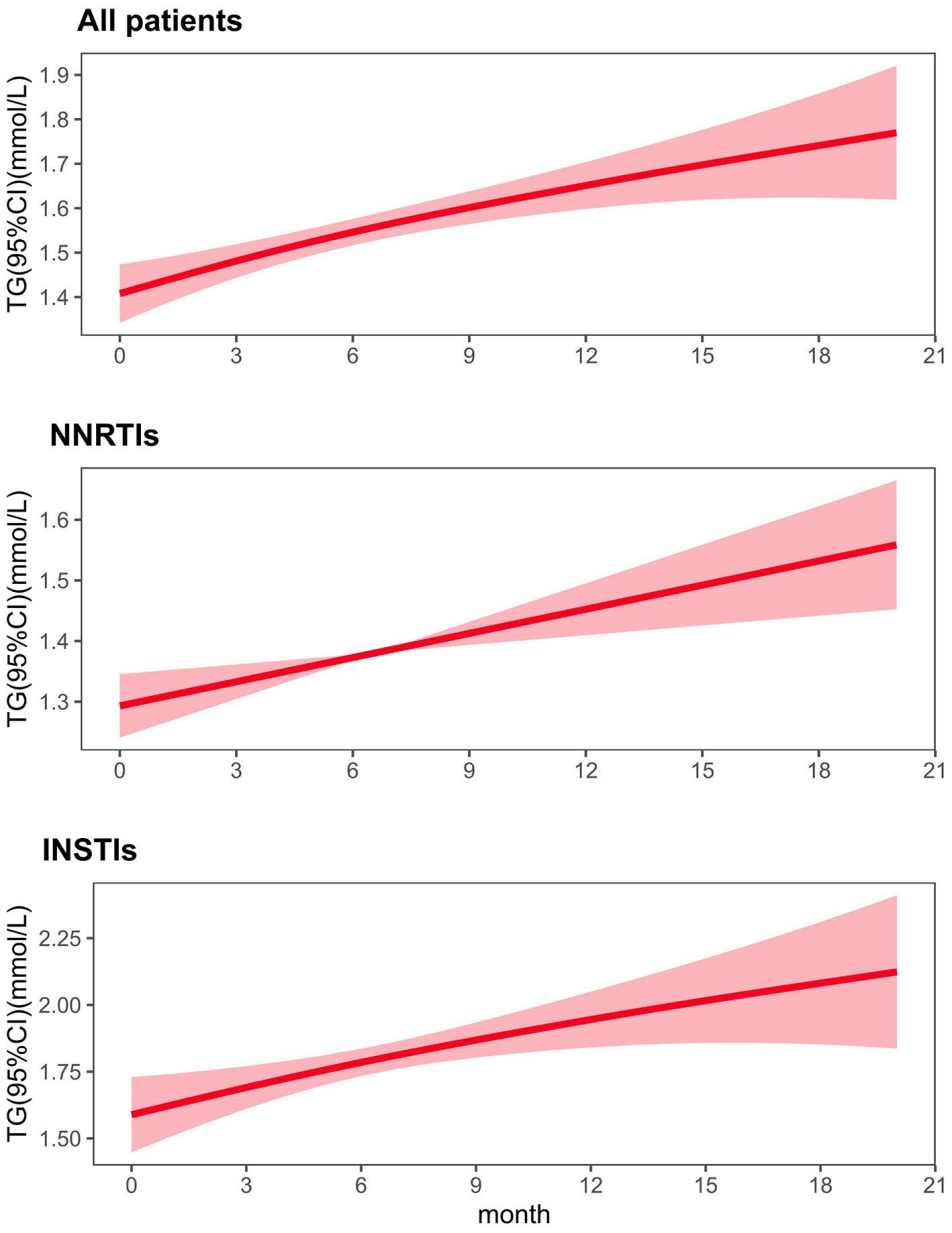
Fitted value changes in triglyceride (lines) over time and corresponding 95% CIs (shaded areas), stratified by types of ART regimen. The results were based on a generalized additive mixed model adjusted for age, gender and BMI. ART antiretroviral therapy, GAMM generalized additive mixed model, CI confidence interval.

Generalized additive mixed models (GAMM) were used to accommodate unbalanced, unequally spaced observations after adjusting for covariates of age, gender and BMI. The GAMM model showed a significant increase in TG values over time in HIV-infected patients receiving ART regimens (Estimate 0.40, SE 0.11, *p* < 0.001; Figure 3).

## Discussion

This study investigated the relationship between two different ART regimens and lipid profiles, and identified risk factors associated with lipid levels using the GLMM in HIV-infected patients. The results showed that HIV-infected patients on INSTIs regimens experienced a larger TG increase than those on the NNRTIs regimens (estimate 0.36, SE 0.14, *p* = 0.008). After adjusting for a wide range of confounders in our analyses, the estimates were robust across sensitivity analyses.

Regardless of whether NNRTIs regimens or INSTIs regimens, the consistency of findings obtained across treatment regimens indicates that the lipid levels and dyslipidemia rates are significantly increased over time in HIV-infected patients receiving ART regimens. These results are consistent with several meta-analyses of randomized controlled trials indicating that ART regimens are significantly associated with an increase in serum lipid levels and increased risk of dyslipidemia (17, 18).

In this longitudinal cohort study, the findings have indicated that the mean values of TC and HDL-C were significantly affected by the ART regimens over time. However, it is important to note that the mean value of TC was higher but HDL-C was lower in the INSTI-treated group than in the NNRTIS-treated group. Although recent findings have established that INSTIs regimens are well tolerated, durable, potent and associated with a lower risk of treatment failure, their effects on serum lipid levels compared to NNRTIs regimens cannot be ignored (19, 20). Atherogenic dyslipidemia, including low HDL-C and high TC, were main cardiovascular risk factors in HIV-infected patients (6, 15, 16). Recent findings have indicated that decreased levels of HDL-C were directly associated with increased CVD risk, and that providing screening and treatment services for ART patients with high cholesterol would reduce the overall CVD risk (21–23). Therefore, recommendations regarding the use of INSTIs regimens should balance their benefits with their potential metabolic harm and CVD risks.

Indeed, with the exception of HDL-C and Lp(a), higher mean values of each serum lipid profiles were observed in HIV patients on INSTIs regimen compared with those on NNRTIs regimen. The present study is inconsistent with earlier studies conducted in Ethiopia, indicating that HIV-infected patients undergoing long-term ART regimen are more likely to have elevated HDL-C as compared with patients undergoing short-term ART regimen (24, 25). Additionally, the present study failed to confirm the findings of the SCRIPT trial, which was significantly associated with elevated Lp(a) levels (14). However, the ARIC study evaluated associations between Lp(a) and incident CVD events in African Americans and Caucasians have shown that race or ethnicity is an important variable influencing Lp(a) levels (26). The discrepancy between the findings of this study and previous studies could be explained by different study populations and limited sample size in this study. Further studies are required to determine the potential clinical implications and the possible associations of specific ART regimens for Lp(a) in HIV-infected patients, given the possible small sample size or short follow-up duration.

Antiretroviral therapy regimens were found to be associated with a high prevalence of dyslipidemia (63.8%) in HIV-infected patients, among which the prevalence of hypercholesterolemia ranging from 8.1 to 16.5%, hypertriglyceridemia ranging from 21.8 to 33.0%, low HDL cholesterol ranging from 42.3 to 63.7% during the 30-month follow-up duration. Previous cross-sectional studies have also reported a high prevalence of dyslipidemia in HIV-infected patients receiving first-line ART (25, 27). However, these studies did not take into account the lipid profile changes over time. In contrast to cross-sectional studies, where it is impossible to assume any causal relationship, this well-controlled cohort study is appropriate to evaluate lipid profile alterations.

In addition, this study found statistically significant differences between NNRTIs and INSTIs groups in the prevalence of TG and high TC/HDL-C ratio. HIV-infected patients on INSTIs regimen were more likely to have high prevalence of hypertriglyceridemia and high TC/HDL-C ratio as compared to those on NNRTIs regimen. A prospective observational study suggested that hypertriglyceridemia (adjusted OR = 3.4; *p* = 0.02) was associated with short-term mortality in HIV-infected patients (28). Another prospective cohort study indicated that the TC/HDL-C ratio (adjusted HR = 5.77, *p* = 0.03) was significantly associated with mortality (29). Based on this, current recommendations for dyslipidemia management suggest the selection of ART regimens with a better lipid profile. Of note, there was a clear turning point in the trend of dyslipidemia rates over time at the 24 months of follow-up periods, which could be attributed to the losses of blood lipids during the longitudinal study. Ideally, the findings of this study should be confirmed in a large-scale, randomized, prospective study with regular monitoring of blood lipids.

In this study, GLMM model showed that older age was significantly associated with higher TC, TG, Lp(a) and TC/HDL-C ratio; higher BMI was significantly associated with higher TG and TC/ HDL-C ratio and lower HDL-C; smoking habit was significantly associated with higher LDL-C, Lp(a) and TC/ HDL-C; lower CD4 count was significantly associated with higher HDL-C and lower TC/HDL-C and the duration of exposure to ART regimen was significantly associated with higher TC and TG. The results from sensitivity analyses also indicated that older age was an independent risk factor for higher TC. In agreement with the results from GLMM model, higher BMI has also been demonstrated to increase TG and TC/HDL-C ratio and decrease HDL-C; lower CD4 counts have also been demonstrated to increase HDL-C and decrease TC/HDL-C ratio by GEE analyses. The present study in NNRTIs-treated patients showed changes in TG values over time, while the consequences of INSTIs-treated patients showed larger increases in TG values. TG values were independently associated with types of ART regimen. Even though there was a wide range of confounding factors and limited sample size in this study, advanced methods and sensitivity analyses were used to obtain robust analysis results. Our findings confirm and extend the results from previous observational studies, which showed that age, gender, BMI, CD4 count, and ART duration were the predictors of unfavorable lipid profiles alterations in patients on ART (24, 25, 30, 31).

Cardiovascular and metabolic complications associated with ART regimens remain reported, and one of the major factors contributing to increased CVD risk in HIV-infected patients receiving ART regimens is dyslipidemia (32–35). In a multivariable regression analysis of CVD risk factors in HIV-infected patients receiving ART regimens, patients with high TC (HR = 1.89, 95%CI 1.27–2.82, *p* = 0.002) and high TG (HR = 1.55, 95%CI 1.02–2.37, *p* = 0.041) were more likely to have CVD events (36). In addition, a cross-sectional study involving HIV-infected patients have shown that raised TC (OR = 1.16, 95%CI 1.00–1.35, *p* = 0.049) and TG (OR = 1.32, 95%CI 1.01–1.73, *p* = 0.044) levels were significantly associated with subclinical atherosclerosis (37). Combining lipid profile alterations with CVD, given the effect of ART regimens on lipid levels and the association with CVD endpoints in HIV-infected patients, future studies are needed to further confirm these findings and determine the optimal lipid management strategies to prevent adverse CVD outcomes.

### Limitations of the study

Several limitations of this study should be noted. This was a purely descriptive study and the results were determined based on a small number of monocentric longitudinal data, which limited the generalizability of the results. This study did not take the potential effects of lipid-lowering agents on patients with dyslipidemia into account, the treating physician did not fully record the application of lipid-lowering drugs at each visit, and our study only estimated the mean lipid profiles of all patients in each follow-up period and comparing to baseline. The methods used might not be sufficient to adequately adjust for these imbalances, thereby affect the findings. Assuming that patients who were more prone to elevated lipid profiles maintain lipid stability with medication, the difference in lipid profile increase between NNRTIs and INSTIs groups would be underestimated. Another important limitation of this study is that unmeasured residual confounders regarding dietary and physical activity could not be ruled out in this observational study, and females comprised only 4.9% (31/633) of all HIV-infected patients, which may have affected the results obtained and be insufficient to detect relevant differences. In addition, due to incomplete data recording in the study and the limited length of the observation periods, which may introduce selection and channeling bias related to missing data into the analysis, more large-scale multicenter studies with sufficient follow-up are warranted to validate the conclusions and optimize ART regimens in the future.

## Conclusion

In conclusion, treatment with both commonly-used ART regimens can increase the mean values of lipid profiles and the risk of dyslipidemia, with INSTIs primarily affecting TC, TG, HDL-C and LDL-C elevations, and NNRTIs were associated with increased TC and HDL-C, but with decreased TC/HDL-C and LDL-C/HDL-C. The rates of abnormal TG and TC/HDL-C are different over time between two ART regimens. The findings of this study indicated that TG values were significantly higher in the INSTIs group than in HIV-infected patients on NNRTIs regimens. Longitudinal TG values are independently associated with the clinical types of ART regimens.

## Data availability statement

The raw data supporting the conclusions of this article will be made available by the authors, without undue reservation.

## Ethics statement

The study was approved by the Institutional Review Board of College of Zhongnan Hospital of Wuhan University (No. 2022122K), and written informed consent was obtained from the study participants. All research procedures were performed in accordance with the approved guidelines and regulations according to the criteria of the Declaration of Helsinki. The Chinese Clinical Trial Registry number of this work is ChiCTR2200059861 (http://www.chictr.org.cn/listbycreater.aspx).

## Author contributions

SL, BW, WL, TC, LD, MZ, and JW made a significant contribution to the work reported, either in terms of conception, study design, execution, data acquisition, analysis and interpretation, or in all these areas; involved in drafting, revising, or critically reviewing the article; gave final approval of the version to be published; gave consent to the journal in which the article was submitted; and agree to be accountable for all aspects of the work.

## Funding

This study was supported by the Translational Medicine and Interdisciplinary Research Joint Fund of Zhongnan Hospital of Wuhan University (grant number ZNJC202201), and the Guiding Project Fund of Hubei Provincial Administration of Traditional Chinese Medicine (grant number ZY2023F047).

## Conflict of interest

The authors declare that the research was conducted in the absence of any commercial or financial relationships that could be construed as a potential conflict of interest.

## Publisher’s note

All claims expressed in this article are solely those of the authors and do not necessarily represent those of their affiliated organizations, or those of the publisher, the editors and the reviewers. Any product that may be evaluated in this article, or claim that may be made by its manufacturer, is not guaranteed or endorsed by the publisher.

## Footnotes

^1^ http://www.chictr.org.cn/listbycreater.aspx
