# Supplementary material for: The effects of ART on the dynamics of lipid profiles in Chinese Han HIV-infected patients: comparison between NRTI/NNRTI and NRTI/INSTI
Source: Front Public Health. 2023 Apr 27;11:1161503. doi: 10.3389/fpubh.2023.1161503 (PMC10174832; doi:10.3389/fpubh.2023.1161503)
Supplement: Supplementary file 1 [file Table_1.docx]

**Appendix Table 1.** **Observations at baseline and all follow**-**up periods for lipid analyses.**

|  | All patients, n (%) | NNRTIs group, n (%) | INSTIs group, n (%) |
| --- | --- | --- | --- |
| Baseline | 546(86.3) | 377(89.3) | 169(80.1) |
| At 6 months | 519(82.0) | 339(80.3) | 180(85.3) |
| At 12 months | 441(69.7) | 301(71.3) | 140(66.4) |
| At 18 months | 313(49.4) | 222(52.6) | 91(43.1) |
| At 24 months | 200(31.6) | 143(33.9) | 57(27.0) |
| At 30 months | 142(22.4) | 107(25.4) | 35(16.6) |

* Lipid profiles is missing for 87 patients at baseline.
