# Supplementary material for: The effects of ART on the dynamics of lipid profiles in Chinese Han HIV-infected patients: comparison between NRTI/NNRTI and NRTI/INSTI
Source: Front Public Health. 2023 Apr 27;11:1161503. doi: 10.3389/fpubh.2023.1161503 (PMC10174832; doi:10.3389/fpubh.2023.1161503)
Supplement: Supplementary file 2 [file Table_2.docx]

**Appendix Table 2. Multivariable analysis of associated factors for lipidemia by generalized linear mixed**-**effects model (GLMM).**

|  | TC | | | TG | | | HDL-C | | |
| --- | --- | --- | --- | --- | --- | --- | --- | --- | --- |
| Fixed Effects | Estimate [95%CI] | Std. Error | *P* values | Estimate [95%CI] | Std. Error | *P* values | Estimate [95%CI] | Std. Error | *P* values |
| Intercept | 3.47[3.78, 4.10] | 0.16 | ***<0.001*** | 1.11[0.77, 1.45] | 0.18 | ***<0.001*** | 1.01[0.91, 1.11] | 0.05 | ***<0.001*** |
| Time | 0.02[0.001, 0.03] | 0.01 | ***0.032*** | 0.02[0.001, 0.04] | 0.01 | ***0.039*** | 0.00[0.00, 0.01] | 0.00 | 0.397 |
| INSTIs vs. NNRTIs | 0.20[-0.05, 0.44] | 0.12 | 0.110 | 0.36[0.10, 0.63] | 0.14 | ***0.008*** | -0.02[-0.10, 0.06] | 0.04 | 0.658 |
| Time: INSTIs vs. NNRTIs | 0.01[-0.01, 0.03] | 0.01 | 0.553 | -0.01[-0.03, 0.02] | 0.01 | 0.494 | 0.00[-0.01, 0.01] | 0.00 | 0.907 |
| Age: 25-44 vs. <25 | 0.23[0.02, 0.44] | 0.11 | ***0.029*** | 0.22[0.01, 0.43] | 0.11 | ***0.042*** | -0.03[-0.09, 0.03] | 0.03 | 0.356 |
| Age: ≥45 vs. <25 | 0.43[0.14, 0.72] | 0.15 | ***0.004*** | 0.09[-0.21, 0.39] | 0.15 | 0.554 | 0.05[-0.04, 0.14] | 0.05 | 0.311 |
| Female vs. Male | 0.30[-0.16, 0.77] | 0.24 | 0.200 | 0.02[-0.46, 0.50] | 0.24 | 0.941 | 0.25[0.11, 0.40] | 0.07 | ***0.001*** |
| BMI: <18.5 vs. 18.5-24.9 | -0.24[-0.53, 0.04] | 0.14 | 0.092 | -0.16[-0.45, 0.13] | 0.15 | 0.281 | 0.06[-0.03, 0.15] | 0.04 | 0.174 |
| BMI: 25-29.9 vs. 18.5-24.9 | 0.15[-0.12, 0.41] | 0.13 | 0.270 | 0.49[0.22, 0.78] | 0.14 | ***<0.001*** | -0.10[-0.18, -0.02] | 0.04 | ***0.017*** |
| BMI: ≥30 vs. 18.5-24.9 | 0.02[-0.67, 0.71] | 0.35 | 0.954 | 0.56[-0.15, 1.27] | 0.36 | 0.124 | -0.13[-0.34, 0.08] | 0.11 | 0.231 |
| Smoking vs. not smoking | 0.17[-0.06, 0.39] | 0.11 | 0.139 | 0.10[-0.13, 0.33] | 0.14 | 0.399 | -0.06[-0.13, 0.01] | 0.04 | 0.097 |
| Drinking vs. not drinking | 0.04[-0.18, 0.25] | 0.11 | 0.747 | 0.10[-0.21, 0.23] | 0.11 | 0.934 | 0.01[-0.05, 0.08] | 0.03 | 0.711 |
| Hypertension vs. not hypertension | -0.39[-0.81, 0.04] | 0.22 | 0.077 | 0.05[-0.39, 0.50] | 0.23 | 0.812 | -0.07[-0.20, 0.07] | 0.07 | 0.317 |
| FPG: ≥7.0 vs. <7.0 | -0.40[-0.83, 0.03] | 0.22 | 0.067 | 0.48[-0.01, 0.96] | 0.25 | 0.054 | -0.13[-0.27, 0.00] | 0.07 | 0.056 |
| CD4 count: <200 vs. ≥200 (cells/mm³) | 0.00[-0.22, 0.21] | 0.11 | 0.969 | -0.16[-0.40, 0.08] | 0.12 | 0.191 | 0.11[0.04, 0.17] | 0.04 | ***0.003*** |
| T lymphocyte count: <955 vs. ≥955 (cells/mm³) | -0.20[-0.39, -0.01] | 0.10 | ***0.037*** | -0.26[-0.47, -0.05] | 0.11 | ***0.017*** | 0.01[-0.05, 0.06] | 0.03 | 0.874 |
| HIV-1 viral load: Positive vs. negative | -0.11[-0.28, 0.06] | 0.09 | 0.187 | 0.11[-0.09, 0.30] | 0.10 | 0.272 | -0.03[-0.08, 0.03] | 0.03 | 0.342 |
| Random Effects |  |  |  |  |  |  |  |  |  |
| Patient ID (random intercept) | 0.35[0.25, 0.48] | 0.06 | ***<0.001*** | 0.22[0.13, 0.39] | 0.06 | ***<0.001*** | 0.03[0.02, 0.05] | 0.01 | ***<0.001*** |
| Time (random slope) | 0.00[0.00, 0.002] | 0.00 | 0.222 | 0.001[0.00, 0.002] | 0.00 | 0.088 | 0.00 | / | / |
|  | LDL-C | | | Lp(a) | | | TC/HDL-C | | |
| Fixed Effects | Estimate [95%CI] | Std. Error | *P* values | Estimate [95%CI] | Std. Error | *P* values | Estimate [95%CI] | Std. Error | *P* values |
| Intercept | 2.35[2.08, 2.61] | 0.14 | ***<0.001*** | 13.54[8.56, 18.52] | 2.53 | ***<0.001*** | 3.99[3.63, 4.35] | 0.18 | ***<0.001*** |
| Time | 0.01[0.00, 0.02] | 0.01 | 0.111 | -0.05[-0.18, 0.08] | 0.07 | 0.457 | 0.01[-0.01, 0.02] | 0.01 | 0.495 |
| INSTIs vs. NNRTIs | 0.06[-0.14, 0.26] | 0.10 | 0.563 | -2.18[-6.39, 2.03] | 2.14 | 0.309 | 0.27[-0.02, 0.55] | 0.15 | 0.066 |
| Time: INSTIs vs. NNRTIs | 0.01[0.00, 0.03] | 0.01 | 0.122 | 0.10[-0.07, 0.27] | 0.09 | 0.247 | 0.01 [-0.02, 0.03] | 0.01 | 0.639 |
| Age: 25-44 vs. <25 | 0.11[-0.07, 0.29] | 0.09 | 0.216 | -0.28[-4.75, 4.19] | 2.27 | 0.903 | 0.30 [0.07, 0.54] | 0.12 | ***0.010*** |
| Age: ≥45 vs. <25 | 0.24[-0.02, 0.49] | 0.13 | 0.065 | 6.55[0.42, 12.69] | 3.12 | ***0.036*** | 0.22[-0.11, 0.55] | 0.17 | 0.187 |
| Female vs. Male | -0.17 [-0.57, 0.23] | 0.20 | 0.406 | -8.75[-18.69, 1.19] | 5.05 | 0.084 | -0.50[-1.02, 0.02] | 0.27 | 0.060 |
| BMI: <18.5 vs. 18.5-24.9 | -0.20[-0.44, 0.05] | 0.12 | 0.110 | -2.58[-8.64, 3.48] | 3.08 | 0.403 | -0.37 [-0.68, -0.05] | 0.16 | ***0.022*** |
| BMI: 25-29.9 vs. 18.5-24.9 | 0.12[-0.10, 0.35] | 0.12 | 0.286 | 0.69[-5.11, 6.49] | 2.95 | 0.233 | 0.52[0.22, 0.82] | 0.15 | ***0.001*** |
| BMI: ≥30 vs. 18.5-24.9 | -0.12[-0.71, 0.47] | 0.30 | 0.684 | -1.35[-16.08, 13.39] | 7.49 | 0.858 | 0.53[-0.25, 1.31] | 0.40 | 0.182 |
| Smoking vs. not smoking | 0.21[0.01, 0.40] | 0.10 | ***0.036*** | 6.02[1.19, 10.86] | 2.46 | ***0.015*** | 0.38[0.13, 0.63] | 0.13 | ***0.003*** |
| Drinking vs. not drinking | -0.03[-0.22, 0.16] | 0.10 | 0.746 | -2.40 [-7.16, 2.35] | 2.42 | 0.320 | -0.06[-0.31, 0.19] | 0.13 | 0.636 |
| Hypertension vs. not hypertension | -0.26[-0.63, 0.11] | 0.19 | 0.170 | -5.55[-14.87, 3.77] | 4.74 | 0.243 | -0.14[-0.63, 0.34] | 0.25 | 0.562 |
| FPG: ≥7.0 vs. <7.0 | -0.46[-0.81, -0.11] | 0.18 | ***0.010*** | -8.31[-12.90, -3.73] | 2.33 | ***<0.001*** | 0.32[-0.18, 0.81] | 0.25 | 0.207 |
| CD4 count: <200 vs. ≥200 (cells/mm³) | -0.05[-0.24, 0.13] | 0.09 | 0.571 | -1.30[-4.05, 1.45] | 1.40 | 0.353 | -0.46[-0.71, -0.21] | 0.13 | ***<0.001*** |
| T lymphocyte count: <955 vs. ≥955 (cells/mm³) | -0.11[-0.27, 0.05] | 0.08 | 0.159 | 0.28[-2.00, 2.54] | 1.15 | 0.238 | -0.23[-0.45, -0.02] | 0.11 | ***0.032*** |
| HIV-1 viral load: Positive vs. negative | -0.12[-0.26, 0.02] | 0.07 | 0.091 | -0.56[-2.34, 1.21] | 0.90 | 0.532 | -0.04[-0.23, 0.15] | 0.10 | 0.662 |
| Random Effects |  |  |  |  |  |  |  |  |  |
| Patient ID (random intercept) | 0.28[0.21, 0.38] | 0.04 | ***<0.001*** | 238.75[198.86, 286.65] | 22.27 | ***<0.001*** | 0.40[0.19, 0.84] | 0.15 | ***0.008*** |
| Time (random slope) | 0.00[0.00, 0.002] | 0.00 | 0.324 | 0.00 | / | / | 0.00 | / | / |

NA = not available. *P* values <0.05 are written in italics.

List of abbreviations: NNRTIs non-nucleoside reverse transcriptase inhibitor, INSTIs integrase strand transfer inhibitors, BMI body mass index, TC total cholesterol, TG triglyceride, HDL-C high-density lipoprotein-cholesterol, LDL-C low-density lipoprotein-cholesterol, Lipoprotein(a) Lp(a), FPG fasting plasma glucose.
