# Supplementary material for: The effects of ART on the dynamics of lipid profiles in Chinese Han HIV-infected patients: comparison between NRTI/NNRTI and NRTI/INSTI
Source: Front Public Health. 2023 Apr 27;11:1161503. doi: 10.3389/fpubh.2023.1161503 (PMC10174832; doi:10.3389/fpubh.2023.1161503)
Supplement: Supplementary file 3 [file Table_3.docx]

**Appendix Table 3.** **Comparison of AIC and BIC of mixed effect multivariable models.**

|  | TC | | TG | | HDL-C | | LDL-C | | Lp(a) | | TC/HDL-C | |
| --- | --- | --- | --- | --- | --- | --- | --- | --- | --- | --- | --- | --- |
|  | AIC | BIC | AIC | BIC | AIC | BIC | AIC | BIC | AIC | BIC | AIC | BIC |
| Model 1 | 1738.127 | 1757.075 | 2222.334 | 2241.277 | 323.896 | 304.948 | 1379.684 | 1398.623 | 6268.317 | 6287.255 | 2340.017 | 2358.965 |
| Model 2 | 1731.461 | 1750.395 | 2221.567 | 2240.496 | 342.912 | 323.979 | 1382.440 | 1401.364 | 6292.630 | 6311.564 | 2323.793 | 2342.726 |
| Model 3 | 1726.765 | 1745.684 | 2205.891 | 2224.805 | 337.937 | 319.017 | 1354.001 | 1372.921 | 6047.182 | 6066.101 | 2312.558 | 2331.477 |
| Model 4 | 1728.320 | 1747.230 | 2205.560 | 2224.465 | 330.542 | 311.632 | 1383.362 | 1402.262 | 6195.964 | 6214.864 | 2306.796 | 2325.706 |
| Model 5 | 1726.147 | 1745.048 | 2205.632 | 2224.527 | 323.168 | 304.268 | 1366.680 | 1385.580 | 6026.658 | 6045.558 | 2306.641 | 2325.541 |
| Model 6 | 795.246 | 810.526 | 899.334 | 914.638 | 6.731 | 22.035 | 716.002 | 731.306 | 2846.941 | 2862.245 | 915.897 | 931.201 |
| Model 7 | 793.550 | 808.782 | 789.215 | 804.412 | 15.508 | 30.765 | 714.610 | 729.866 | 2821.726 | 2836.983 | 910.329 | 925.586 |

Model 1 – ART only as variables.

Model 2 – ART, age, and sex as variables.

Model 3 – ART, age, sex and body mass index as variables.

Model 4 – ART, age, sex, body mass index, drinking and smoking as variables.

Model 5 – ART, age, sex, body mass index, fasting plasma glucose, drinking, smoking and hypertension as variables.

Model 6 – ART, age, sex, body mass index, CD4 count, T lymphocyte count and HIV-1 viral load as variables.

Model 7 – ART, age, sex, body mass index, fasting plasma glucose, drinking, smoking, hypertension, CD4 count, T lymphocyte count and HIV-1 viral load as variables.

List of abbreviations: ART antiretroviral therapy, AIC Akaike information criterion, BIC Bayesian information criteria, TC total cholesterol, TG triglyceride, HDL-C high-density lipoprotein-cholesterol, LDL-C low-density lipoprotein-cholesterol, Lipoprotein(a) Lp(a), TC/HDL-C ratio total cholesterol/high-density lipoprotein-cholesterol ratio.
