# Supplementary material for: The effects of ART on the dynamics of lipid profiles in Chinese Han HIV-infected patients: comparison between NRTI/NNRTI and NRTI/INSTI
Source: Front Public Health. 2023 Apr 27;11:1161503. doi: 10.3389/fpubh.2023.1161503 (PMC10174832; doi:10.3389/fpubh.2023.1161503)
Supplement: Supplementary file 4 [file Table_4.docx]

**Appendix Table 4. Sensitivity analysis of associated factors for lipidemia by generalized estimating equation (GEE).**

|  | TC | | TG | | HDL-C | |
| --- | --- | --- | --- | --- | --- | --- |
| Risk factors | Exp(B) [95%CI] | *P* values | Exp(B) [95%CI] | *P* values | Exp(B) [95%CI] | *P* values |
| INSTIs vs. NNRTIs | 1.05[0.77, 1.43] | 0.750 | 1.67[1.05, 2.67] | ***0.031*** | 0.97[0.87, 1.08] | 0.572 |
| Age: 25-44 vs. <25 | 1.24[1.03, 1.50] | ***0.024*** | 1.20[0.94, 1.52] | 0.135 | 0.98[0.92, 1.04] | 0.465 |
| Age: ≥45 vs. <25 | 1.51[1.18, 1.92] | ***0.001*** | 1.02[0.75, 1.37] | 0.921 | 1.04[0.96, 1.14] | 0.325 |
| Female vs. Male | 1.46[0.97, 2.21] | 0.072 | 1.07[0.73, 1.59] | 0.717 | 1.33[1.12, 1.58] | ***0.001*** |
| BMI: <18.5 vs. 18.5-24.9 | 0.79[0.60, 1.05] | 0.103 | 0.82[0.65, 1.04] | 0.103 | 1.07[0.97, 1.18] | 0.203 |
| BMI: 25-29.9 vs. 18.5-24.9 | 1.22[0.98, 1.52] | 0.077 | 1.61[1.09, 2.38] | ***0.016*** | 0.92[0.86, 0.98] | ***0.015*** |
| BMI: ≥30 vs. 18.5-24.9 | 0.97[0.52, 1.83] | 0.931 | 1.85[1.10, 3.11] | ***0.021*** | 0.88[0.80, 0.96] | ***0.006*** |
| Smoking vs. not smoking | 1.18[0.93, 1.50] | 0.165 | 1.17[0.92, 1.49] | 0.198 | 0.94[0.88, 1.00] | 0.070 |
| Drinking vs. not drinking | 1.05[0.85, 1.30] | 0.659 | 1.00[0.80, 1.25] | 0.991 | 1.02[0.96, 1.08] | 0.549 |
| Hypertension vs. not hypertension | 0.69[0.42, 1.13] | 0.142 | 1.04[0.67, 1.61] | 0.859 | 0.93[0.81, 1.08] | 0.362 |
| FPG: ≥7.0 vs. <7.0 | 0.69[0.44, 1.09] | 0.109 | 1.78[0.86, 3.69] | 0.123 | 0.87[0.79, 0.95] | ***0.004*** |
| CD4 count: <200 vs. ≥200 (cells/mm³) | 0.96[0.76, 1.22] | 0.755 | 0.77[0.61, 0.99] | ***0.038*** | 1.11[1.03, 1.20] | ***0.010*** |
| T lymphocyte count: <955 vs. ≥955 (cells/mm³) | 0.86[0.70, 1.06] | 0.147 | 0.73[0.61, 0.87] | ***0.001*** | 1.02[0.96, 1.09] | 0.553 |
| HIV-1 viral load: Positive vs. negative | 0.89[0.73, 1.09] | 0.253 | 1.06 [0.84, 1.33] | 0.636 | 1.00[0.94, 1.06] | 0.874 |
|  | LDL-C | | Lp(a) | | TC/HDL-C | |
| Risk factors | Exp(B) [95%CI] | *P* values | Exp(B) [95%CI] | *P* values | Exp(B) [95%CI] | *P* values |
| INSTIs vs. NNRTIs | 0.86[0.66, 1.14] | 0.300 | 6.54[0.01, 8.55] | 0.581 | 1.12[0.80, 1.57] | 0.501 |
| Age: 25-44 vs. <25 | 1.12[0.95, 1.33] | 0.186 | 0.59[0.01, 27.60] | 0.788 | 1.30 [1.06, 1.59] | ***0.012*** |
| Age: ≥45 vs. <25 | 0.11[-0.07, 0.29] | ***0.024*** | 7.05[0.72, 14.44] | 0.061 | 1.25[0.95, 1.66] | 0.115 |
| Female vs. Male | 0.87 [0.63, 1.21] | 0.418 | 0.00[0.00, 0.04] | ***0.003*** | 0.59[0.39, 0.88] | ***0.010*** |
| BMI: <18.5 vs. 18.5-24.9 | 0.83 [0.66, 1.03] | 0.096 | 0.08[0.00, 4.30] | 0.217 | 0.66 [0.52, 0.83] | ***<0.001*** |
| BMI: 25-29.9 vs. 18.5-24.9 | 1.17[0.92, 1.47] | 0.194 | 2.56[0.11, 6.95] | 0.759 | 1.64[1.28, 2.08] | ***<0.001*** |
| BMI: ≥30 vs. 18.5-24.9 | 0.84[0.48, 1.47] | 0.537 | 0.46[0.00, 54.61] | 0.752 | 1.64[0.78, 3.45] | 0.189 |
| Smoking vs. not smoking | 1.22[1.02, 1.46] | ***0.034*** | 5.93[1.28, 11.62] | ***0.041*** | 1.50[1.15, 1.95] | ***0.002*** |
| Drinking vs. not drinking | 0.98[0.81, 1.18] | 0.796 | 0.09 [0.00, 12.06] | 0.332 | 0.94[0.74, 1.18] | 0.581 |
| Hypertension vs. not hypertension | 0.77[0.47, 1.26] | 0.301 | 0.00[0.00, 6.17] | 0.354 | 0.87[0.48, 1.56] | 0.635 |
| FPG: ≥7.0 vs. <7.0 | 0.64[0.42, 0.97] | ***0.036*** | 0.00[0.00, 0.17] | ***0.014*** | 1.46[0.77, 2.78] | 0.250 |
| CD4 count: <200 vs. ≥200 (cells/mm³) | 0.95[0.79, 1.16] | 0.639 | 1.19[0.01, 5.32] | 0.948 | 0.57 [0.43, 0.77] | ***<0.001*** |
| T lymphocyte count: <955 vs. ≥955 (cells/mm³) | 0.95[0.79, 1.12] | 0.524 | 0.16[0.00, 13.78] | 0.417 | 0.76[0.61, 0.95] | ***0.014*** |
| HIV-1 viral load: Positive vs. negative | 0.87[0.73, 1.03] | 0.105 | 1.57[0.04, 4.03] | 0.804 | 0.86[0.70, 1.06] | 0.154 |

NA = not available. *P* values <0.05 are written in italics.

List of abbreviations: NNRTIs non-nucleoside reverse transcriptase inhibitor, INSTIs integrase strand transfer inhibitors, BMI body mass index, TC total cholesterol, TG triglyceride, HDL-C high-density lipoprotein-cholesterol, LDL-C low-density lipoprotein-cholesterol, Lipoprotein(a) Lp(a), FPG fasting plasma glucose.
