# Supplementary material for: The effects of ART on the dynamics of lipid profiles in Chinese Han HIV-infected patients: comparison between NRTI/NNRTI and NRTI/INSTI
Source: Front Public Health. 2023 Apr 27;11:1161503. doi: 10.3389/fpubh.2023.1161503 (PMC10174832; doi:10.3389/fpubh.2023.1161503)
Supplement: Supplementary file 5 [file Presentation_1.pdf]

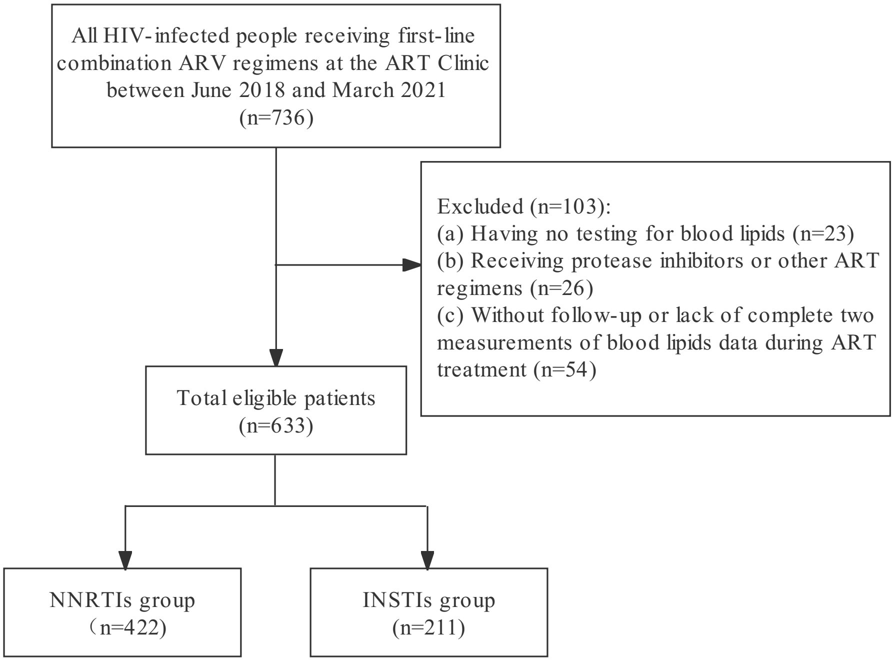
All HIV-infected people receiving first-line combination ARV regimens at the ART Clinic between June 2018 and March 2021 (n=736)
Excluded (n=103):
(a) Having no testing for blood lipids (n=23)
(b) Receiving protease inhibitors or other ART regimens (n=26)
(c) Without follow-up or lack of complete two measurements of blood lipids data during ART treatment (n=54)
Total eligible patients (n=633)
NNRTIs group （n=422)
INSTIs group (n=211)
