# Supplementary material for: The effects of ART on the dynamics of lipid profiles in Chinese Han HIV-infected patients: comparison between NRTI/NNRTI and NRTI/INSTI
Source: Front Public Health. 2023 Apr 27;11:1161503. doi: 10.3389/fpubh.2023.1161503 (PMC10174832; doi:10.3389/fpubh.2023.1161503)
Supplement: Supplementary file 6 [file Data_Sheet_1.docx]

**Figure 1.** (A) The mean values of total cholesterol during follow-up period with NNRTIs vs. INSTIs. (B) Triglycerides throughout the follow-up period. (C) HDL-cholesterol. (D) LDL-cholesterol. (E) Lipoprotein(a). (F) TC/HDL-C ratio. (G) TG/HDL-C ratio. (G) LDL-C/HDL-C ratio.

**Figure 2.** (A) The prevalence of abnormal total cholesterol during follow-up period with NNRTIs vs. INSTIs. (B) Triglycerides throughout the follow-up period. (C) HDL-cholesterol. (D) LDL-cholesterol. (E) Lipoprotein(a). (F) TC/HDL-C ratio.

**Figure 3.** Fitted value changes in triglyceride (lines) over time and corresponding 95% CIs (shaded areas), stratified by types of ART regimen. The results were based on a generalized additive mixed model adjusted for age, gender and BMI. List of abbreviations: ART antiretroviral therapy, GAMM generalized additive mixed model, CI confidence interval.

**Appendix Figure 1.** Selection of the study population according to inclusion and exclusion criteria.
